# Characterization of Two *Streptomyces* Enzymes That Convert Ferulic Acid to Vanillin

**DOI:** 10.1371/journal.pone.0067339

**Published:** 2013-06-28

**Authors:** Wenwen Yang, Hongzhi Tang, Jun Ni, Qiulin Wu, Dongliang Hua, Fei Tao, Ping Xu

**Affiliations:** 1 State Key Laboratory of Microbial Metabolism, School of Life Sciences and Biotechnology, Shanghai Jiao Tong University, Shanghai, People’s Republic of China; 2 Key Laboratory for Biomass Gasification Technology of Shandong Province, Energy Research Institute of Shandong Academy of Sciences, Jinan, People’s Republic of China; Belgian Nuclear Research Centre SCK/CEN, Belgium

## Abstract

Production of flavors from natural substrates by microbial transformation has become a growing and expanding field of study over the past decades. Vanillin, a major component of vanilla flavor, is a principal flavoring compound used worldwide. *Streptomyces* sp. strain V-1 is known to be one of the most promising microbial producers of natural vanillin from ferulic acid. Although identification of the microbial genes involved in the biotransformation of ferulic acid to vanillin has been previously reported, purification and detailed characterization of the corresponding enzymes with important functions have rarely been studied. In this study, we isolated and identified 2 critical genes, *fcs* and *ech*, encoding feruloyl-CoA synthetase and enoyl-CoA hydratase/aldolase, respectively, which are involved in the vanillin production from ferulic acid. Both genes were heterologously expressed in *Escherichia coli*, and the resting cell reactions for converting ferulic acid to vanillin were performed. The corresponding crucial enzymes, Fcs and Ech, were purified for the first time and the enzymatic activity of each purified protein was studied. Furthermore, Fcs was comprehensively characterized, at an optimal pH of 7.0 and temperature of 30°C. Kinetic constants for Fcs revealed the apparent *K*_m_, *k*_cat_, and *V*_max_ values to be 0.35 mM, 67.7 s^−1^, and 78.2 U mg^−1^, respectively. The catalytic efficiency (*k*_cat_/*K*_m_) value of Fcs was 193.4 mM^−1^ s^−1^ for ferulic acid. The characterization of Fcs and Ech may be helpful for further research in the field of enzymatic engineering and metabolic regulation.

## Introduction

Vanillin (4-hydroxy-3-methoxybenzaldehyde) is one of the most important flavoring agents used in the world. It is widely used in foods, beverages, perfumes, and pharmaceuticals owing to its unique and irreplaceable flavor [1]. Its annual worldwide consumption is estimated to be over 16,000 tons [2]. Natural vanillin extracted from vanilla pods provides only about 0.25% (40 tons of 16,000 tons) of vanillin sold in the market, whereas the remainder is mostly produced through the chemical synthesis of lignin or fossil hydrocarbons [3]. However, natural vanillin has a market price that is 300-times higher than the synthetic “unnatural” vanillin, which is sold for approximately $15 per kilogram [4]. Alternatively, vanillin produced by biotechnological approaches in microbial systems is classified as natural vanillin by the European and US food legislations [5]. Because of the increasing concerns for “natural” and “healthy” products among consumers worldwide, the microbial production of natural vanillin has become one of the major points of research in this field.

Ferulic acid (4-hydroxy-3-methoxycinnamic acid) is an extremely abundant hydroxycinnamic acid found in cell walls of cereals, woods, and sugar beet [1], [6]. As ferulic acid is an economical and natural source, it is one of the potential vanillin precursors in the biotransformation process. In recent decades, vanillin production has been reported for various microorganisms, including *Pseudomonas* sp. HR199 [7], *Amycolatopsis* sp. ATCC 39116 [8], [9], *Amycolatopsis* sp. HR167 [10], *Sphingomonas paucimobilis* SYK-6 [11], *Rhodococcus* strains [12], *Pseudomonas fluorescens* AN103 [13], and *Streptomyces setonii* [4]. Such vanillin-producing strains generally use a consistent coenzyme A-dependent, non-*β*-oxidative pathway for ferulic acid bioconversion, which involves 2 genes *fcs* and *ech*, encoding feruloyl-CoA synthetase (Fcs) and enoyl-CoA hydratase/aldolase (Ech), respectively [7]. However, purification and comprehensive characterization of the functional enzymes involved in this biotransformation have rarely been elucidated. *Streptomyces* sp. strain V-1 for the vanillin production has previously been isolated [14]. When 8% vanillin-absorbent resin DM11 (wet w:v) was applied, strain V-1 was able to transform 45 g L^−1^ ferulic acid to produce 19.2 g L^−1^ vanillin within a 55-h fed-batch fermentation process [14]. *Streptomyces* sp. strain V-1 is known to be one of the best vanillin-producing strains, giving the highest production with a 54.5% molar yield of vanillin from ferulic acid [14], which is lower than the >70% molar yield of vanillin produced by two other highly productive strains, *Amycolatopsis* sp. strains HR167 and ATCC 39116 [9], [10], [15]. Previous research on strain V-1 mainly focused on the biotransformation process and enhancing vanillin productivity, whereas less attention was paid to the research at the molecular and enzymatic level.

In this study, we cloned and identified 2 genes, *fcs* and *ech*, which are responsible for vanillin production from ferulic acid in *Streptomyces* sp. strain V-1. Both genes were heterologously expressed and purified from recombinant *Escherichia coli*. Fcs, in particular, was biochemically characterized in detail.

## Materials and Methods

### Chemicals

Ferulic acid, vanillin, coenzyme A hydrate (CoA), and adenosine 5′-triphosphate disodium salt hydrate (ATP) were all purchased from Sigma-Aldrich (St. Louis, MO, USA). Isopropyl-*β*-d-1-thiogalactopyranoside (IPTG) was purchased from Merck (Darmstadt, Germany). All other commercially available chemicals were of analytical grade or chromatographically pure.

### Plasmids, Bacterial Strains and Culture Conditions

The bacterial strains and plasmids used in this study are listed in Table 1. *Streptomyces* sp. V-1 (CCTCC M 206065) was cultivated at 30°C in the seed medium, which contained 10 g L^−1^ glucose, 5 g L^−1^ yeast extract, 10 g L^−1^ peptone, 5 g L^−1^ beef extract, and 2 g L^−1^ NaCl (pH 7.0) as previously described [14]. *E. coli* BL21 (DE3) (TransGen, Beijing, China) was used as the host strain in cloning and expressing experiments. The *E. coli* strains were grown in lysogenic broth (LB) medium containing 100 mg L^−1^ ampicillin or 50 mg L^−1^ kanamycin at 37°C.

**Table 1:** Bacterial strains and plasmids used in this study.

Strain and plasmid	Description	Source
**Strain**		
*Streptomyces* sp. V-1	Wild-type (CCTCC M 206065)	Laboratory stock
*E. coli* BL21 (DE3)	F^−^, *ompT*, *hsdS*(r_B_^−^ m_B_^−^), *gal*, *dcm*(DE3)	TransGen
*E. coli* BL21 (pETDuet-1)	BL21 (DE3) containing plasmid pETDuet-1	This work
*E. coli* BL21 (pETDuet-*ech*-*fcs*)	BL21 (DE3) containing plasmid pETDuet-*ech*-*fcs*	This work
*E. coli* BL21 (pET28a)	BL21 (DE3) containing plasmid pET28a	This work
*E. coli* BL21 (pET28a-*ech*)	BL21 (DE3) containing plasmid pET28a-*ech*	This work
*E. coli* BL21 (pET28a-*fcs*)	BL21 (DE3) containing plasmid pET28a-*fcs*	This work
**Plasmid**		
pMD18-T	Cloning vector, Amp^r^, *lacZ*, ColE1 ori	TaKaRa
pETDuet-1	Amp^r^, *lacZ*, T7 promoter	Novagen
pETDuet-*ech*-*fcs*	pETDuet-1 carrying *ech* and *fcs* genes of V-1	This work
pET28a (+)	Kan^r^, *lacZ*, T7 promoter	Novagen
pET28a-*ech*	pET28a (+) carrying *ech* of V-1	This work
pET28a-*fcs*	pET28a (+) carrying *fcs* of V-1	This work

### Cloning of 2 Critical Genes *fcs* and *ech*

DNA manipulation and transformation were performed according to the standard procedures [16]. Genomic DNA was extracted from *Streptomyces* sp. V-1 by using a bacterial genomic DNA extraction kit (QIAGEN, Hilden, Germany).

According to the previous reports [7], [10], [11], a higher homology with the deduced amino acid alignment from *ech* was found, while no significant similarity was found to *fcs*. Therefore, we firstly isolated gene *ech* from *Streptomyces* sp. V-1. We carried out the sequence alignment and comparison using the clustalX software [17]. We designed the degenerate primers (P1 and P2, listed in Table 2) according to the most conserved Ech regions in *Pseudomonas* sp. HR199 [7], *Sphingomonas paucimobilis* SYK-6 [11], *Pseudomonas putida* WCS358 [18], *Pseudomonas fluorescens* AN103 [13] and *Amycolatopsis* sp. HR167 [10] (Figure S1). With the genomic DNA of V-1 as the template, PCR was carried out with LA Taq DNA polymerase, 2×GC buffer I, and 2.5 mM deoxynucleoside triphosphates (TaKaRa Bio Inc., Shiga, Japan). The PCR cycling conditions were as follows: 94°C for 3 min followed by 35 cycles of 94°C for 30 s, 55.0∼70.0°C for 30 s, and 72°C for 15 s, and a final extension at 72°C for 5 min. The target gene fragment of approximately 260 bp was sequenced by Invitrogen Corp. (Shanghai, China).

**Table 2:** Primers used for PCR analysis.

Primer	Sequence (5′-3′)	Recombinant plasmid
P1	AGCCCRACBCTSAAC	
P2	GCCGAAGCACCAGCC	
SP1	AGCCCGACCCTGAACGACGAGATGGTGC	
SP2	GACGACCGCTGCCGAGTGCTGGTGCT	
SP3	CGTGCAGATCAAGGTGNNNNNNNNNAGCG	
P3	ATGAGCACAGCGGTCGGCAAC	pMD18T-*ech*
P4	CTACTTCTCCGGGTCGAAGGCGCT	
P5	ATGCGCAACCAGGGTCTGGGC	pMD18T-*fcs*
P6	TCAGCCGAAGCGGCGGCGGACCTC	
P7	GGGAATTCCATATGCGCAACCAGGGTCTGGGC	pET28a-*ech*
P8	CCGCTCGAGTCAGCCGAAGCGGCGGCGGACCTCGCC	
P9	GGGAATTCCATATGCGCAACCAGGGTCTGGGC	pET28a-*fcs*
P10	CCGCTCGAGTCAGCCGAAGCGGCGGCGGACCTCGCC	
P11	CATGCCATGGGCATGAGCACAGCGGTCGGCAACGGG	pETDuet-*ech*
P12	CCCAAGCTTCTACTTCTCCGGGTCGAAGGCGCTCAG	
P13	GGGAATTCCATATGCGCAACCAGGGTCTGGGC	pETDuet-*fcs*
P14	CCGCTCGAGTCAGCCGAAGCGGCGGCGGACCTCGCC	

Symbols for degenerate primers: R = A/G, M = A/C, S = C/G, W = A/T, Y = C/T, K = T/G, H = A/T/C, B = G/T/C, D = A/G/T, V = A/G/C, N = A/T/G/C.

Secondly, we applied a modified method for chromosome walking known as self-formed adaptor PCR (SEFA-PCR) [19]. The obtained gene fragment was extended with 3 specific primers SP1, SP2, and SP3 (Table 2), respectively. The design of the specific primers, PCR conditions and procedures were all strictly performed as reported previously [19]. A 750-bp gene fragment was identified, and it showed the highest homology with enoyl-CoA hydratase/aldolase of *Amycolatopsis* sp. HR167 (99% identity) [10]. We re-designed a pair of primers (P3 and P4, listed in Table 2) according to the sequence of *ech* from *Amycolatopsis* sp. HR167 [10]. The PCR procedures were the same as those described above, and the target gene fragment was ligated to pMD18-T (TaKaRa) and sequenced. Finally, we isolated the full-length gene *ech* from *Streptomyces* sp. V-1.

Meanwhile, we also designed a pair of primers (P5 and P6, listed in Table 2) according to the sequence of *fcs* from *Amycolatopsis* sp. HR167 [10]. And PCR was performed according to the same procedure as that of *ech*, with the genomic DNA of V-1 as the template in PCR amplification. Accordingly, the full-length gene *fcs* from *Streptomyces* sp. V-1 could be obtained.

### Construction of Plasmids for the Two Genes *fcs* and *ech*

Each of the amplified products *ech* and *fcs* was subcloned into the pET-28a vector (Novagen, Merck KGaA, Darmstadt, Germany) with the primer pairs P7 and P8, P9 and P10 (listed in Table 2) to produce two recombinant plasmids pET28a-*ech* and pET28a-*fcs*, respectively. The resulting plasmid was then transformed into *E. coli* BL21 (DE3) for expression.

In addition, for functional identification, the *fcs* gene was ligated into the *Nde*I and *Xho*I sites of Multiple Cloning Site II (MCSII), while the *ech* gene was ligated into the *Nco*I and *Hin*dIII sites of MCSI of a pETDuet-1 vector (Novagen), thus making a recombinant plasmid pETDuet-*ech*-*fcs*. The resulting plasmid was then transformed into *E. coli* BL21 (DE3) for co-expression.

### Purification of his_6_-tagged Fcs

The *E. coli* harboring corresponding plasmid pET28a-*fcs* was grown at 37°C in 100 mL of LB medium containing 50 mg L^−1^ kanamycin. When the OD_600 nm_ reached 0.6∼0.8, IPTG was added to the culture at a final concentration of 0.2 mM, and the culture was incubated for 8 h at 16°C for over-expression.

The induced *E. coli* cells were washed and resuspended in buffer A (25 mM Tris-HCl, pH 8.0, 300 mM NaCl, and 20 mM imidazole). The protease inhibitor phenylmethanesulfonyl fluoride was added to the cell suspension at a final concentration of 0.1 mM. The cells were then lysed by sonication, and the soluble fractions were obtained by centrifugation at 11,000×*g* at 4°C for 30 min. All steps for protein purification were performed at 4°C by using an AKTA purifier chromatography system (GE Healthcare Bio-Sciences Corp., Piscataway, NJ, USA). The crude cell extracts were loaded onto a HisTrap FF column (5 mL, GE Healthcare), pre-equilibrated with buffer A, and eluted with a linear gradient from 0% to 100% with buffer B (25 mM Tris-HCl, pH 8.0, 300 mM NaCl, and 500 mM imidazole). The active protein solution was then applied to a HiTrap Q Sepharose XL column (1 mL, GE Healthcare), pre-equilibrated with buffer C (20 mM Tris-HCl, pH 9.0), and eluted with a 30-mL linear gradient from 0% to 100% with buffer D (20 mM Tris-HCl, 1 M NaCl, pH 9.0) for anion exchange chromatography. Active fractions were desalted by a HiTrap Desalting column (5 mL, GE Healthcare) with buffer C. Finally, the purified protein Fcs was obtained and concentrated to 0.67 mg mL^−1^, and then stored at −80°C until further use.

The protein concentration was determined according to the Bradford method [20]. Sodium dodecyl sulfate-polyacrylamide gel electrophoresis (SDS-PAGE) was performed using a 12.5% gel in a MiniProtein III electrophoresis cell (Bio-Rad, Hercules, CA, USA) [21]. The gel was stained with Coomassie brilliant blue R250.

### Purification of his_6_-tagged Ech

The crude cell extracts of the induced recombinant strain *E. coli* BL21 (pET28a-*ech*) were prepared according to the method described above. The extracts were loaded onto a HisTrap FF column (5 mL, GE Healthcare), pre-equilibrated with buffer A, and eluted with a linear gradient from 0% to 100% with buffer B. Active fractions were desalted by a HiTrap Desalting column (5 mL, GE Healthcare) with buffer C. Therefore, the purified protein Ech was obtained and stored at −80°C for further use.

### Enzymatic Assay

The enzymatic assay of Fcs was performed according to a previous report [10]. The reaction mixture (1 mL) contained 100 mM potassium phosphate buffer (pH 7.0), 2.5 mM MgCl_2_, 0.7 mM ferulic acid, 2 mM ATP, 0.4 mM coenzyme A, and 1.34 µg of the purified protein Fcs. The activity assay was started by the addition of ATP, and the initial absorbance increase due to the formation of feruloyl-CoA (*ε*  = 10 cm^2^ µmol^−1^) was measured at 345 nm using a UV-visible 2550 spectrophotometer (Shimadzu, Kyoto, Japan).

### Effects of pH, Temperature, and Metal Salts on the Enzyme Activity

The Fcs activity was assayed according to the above described method after 10-min incubation at 30°C in 100 mM K_2_HPO_4_-KH_2_PO_4_ buffers, with the pH ranging from 5.0 to 11.0. The temperatures ranging from 16°C to 50°C were chosen for the determination of Fcs activity. The assay was carried out in 100 mM potassium phosphate buffer (pH 7.0) after 10-min incubation at the corresponding determination temperature. To assess the effect of metal salts on the Fcs activity, 10 different metal salts (Mg^2+^, Mn^2+^, Co^2+^, Ni^2+^, Zn^2+^, Cu^2+^, Ca^2+^, Fe^2+^, MoO_4_^2−^, and WO_4_^2−^) were applied in the reaction mixture at a final concentration of 2.5 mM, with reaction conditions maintained as outlined above. The initial absorbance increase due to the formation of feruloyl-CoA (*ε*  = 10 cm^2^ µmol^−1^) was measured at 345 nm after the addition of ATP. Each experiment was run in triplicate.

### Kinetic Study

The kinetic parameters of Fcs were determined in a 100 mM K_2_HPO_4_-KH_2_PO_4_ buffer (pH 7.0) at 30°C in the presence of Mg^2+^, CoA, and ATP. The *K*_m_ and *V*_max_ values for ferulic acid were determined with varied concentrations of ferulic acid ranging from 0.175 mM to 0.7 mM. Each sample at a specific concentration of ferulic acid was run in triplicate. *K*_m_ and *V*_max_ values were obtained by calculating the slope (*K*_m_/*V*_max_) and the *x* intercept (−1/*K*_m_) through Michaelis-Menten plots and Lineweaver-Burk reciprocal plots.

### Resting Cell Transformation

Resting cells of the recombinant *E. coli* BL21 (pETDuet-*ech*-*fcs*) were prepared as reported previously [7], [10] with minor modification. The recombinant strain *E. coli* BL21 (pETDuet-*ech*-*fcs*) was grown at 37°C in 100 mL of LB medium containing 100 mg L^−1^ ampicillin. When the OD_600 nm_ reached 0.6∼0.8, IPTG was added to the culture at a final concentration of 0.2 mM. After 8 h of incubation at 16°C, cells were harvested by centrifugation (3,500×*g*, 15 min) at 4°C, washed twice with 10 mM PBS buffer (137 mM NaCl, 2.7 mM KCl, 10 mM Na_2_HPO_4_, and 2 mM KH_2_PO_4_, pH 7.4), and resuspended in the same buffer to OD_600 nm_  = 30.

Ferulic acid was dissolved in dimethyl sulfoxide to prepare a storage solution of 400 g L^−1^, which was then added into the reaction mixture at a specific final concentration. Reactions were performed in 250-mL flasks containing 25 mL cell suspensions at 30°C, and flasks were shaken at 200 rpm. At regular time intervals, 1-mL samples were removed from the flasks for gas chromatography (GC) analysis to detect the transformation of ferulic acid and production of vanillin.

### Analytical Method

For functional identification of the *fcs* and *ech* genes, we used a GC system to determine the substrate decrease or the product formation. The pH value of the sample was adjusted to below 2 with 6 M HCl and extracted with butyl acetate (1∶1, v:v). Analysis was performed using an Agilent Technologies 7890A GC system (Santa Clara, CA, USA), equipped with a 30-m HP-5 capillary column (30 m×0.320 mm×0.25 µm, Agilent Technologies). The temperatures of the injector and flame ionization detector were both 280°C. The column oven temperature was maintained at 140°C for 2 min, then raised to 220°C at a rate of 15°C min^−1^, and held for 2 min.

### Nucleotide Sequence Accession Number

The nucleotide and amino acid sequences of *fcs* and *ech* reported in this study have been submitted to the GenBank sequence databases under accession no. KC847405 and KC847406, respectively.

## Results

### Cloning of the Genes for Vanillin Production from Ferulic Acid

Conventional PCR strategies, making use of degenerate primers of Ech and Fcs, followed by a modified method for chromosome walking (SEFA-PCR) [19], were performed to isolate the genes responsible for vanillin production from ferulic acid in *Streptomyces* sp. strain V-1. Two gene fragments of 1,476 bp and 864 bp, representing *fcs* and *ech*, respectively, were isolated. The G+C contents of *fcs* and *ech* were 74.1% and 70.3%, respectively. The *fcs* gene used GTG as the start codon and TGA as the stop codon, whereas *ech* used ATG and TAG.

As for the localization of *fcs* and *ech*, it was reported that the translational start codon GTG of *fcs* overlapped with the stop codon TAG of *ech* in *Amycolatopsis* sp. strain HR167 [10]. For *Streptomyces* sp. strain V-1, a clear band of the right size (2,339 bp) was observed on the agarose gel (data not shown) by PCR amplication with the primer pair P3 (the forward primer of *ech*) and P6 (the reverse primer of *fcs*). Through sequencing analysis, it was found that the *ech* and *fcs* genes were located just in the same way as that reported in *Amycolatopsis* sp. strain HR167 [10]. The stop codon of the upstream gene *ech* overlaps the start codon of the downstream gene *fcs*, which is an indication for “translational coupling”, a phenomenon allowing co-regulated gene expression and stochiometric maintenance of gene products [22].

### Heterologous Expression and Functional Identification of *fcs* and *ech*

The enzyme activity of the gene product of *fcs* from *Streptomyces* sp. V-1 was identified by the increase in the initial absorbance at 345 nm of the reaction mixture containing the cell extracts of recombinant *E. coli* BL21 (pET28a-*fcs*). Furthermore, the decrease in the concentration of ferulic acid in this reaction mixture was also detected by high performance liquid chromatography (HPLC) (data not shown).

**Figure 1:**
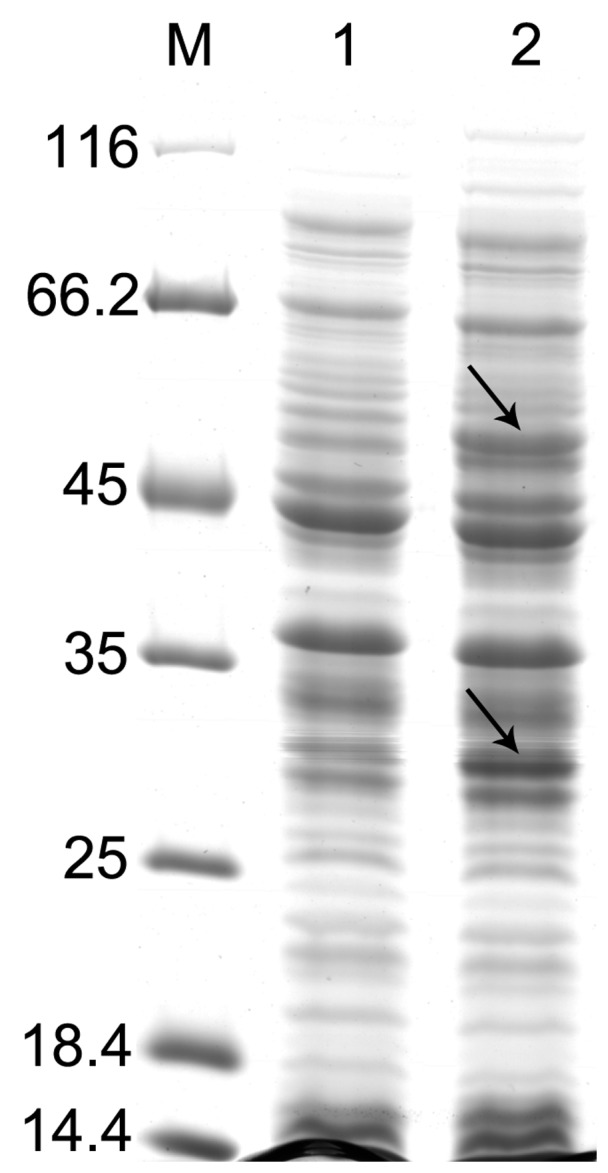
SDS-PAGE of over-expressed *fcs* and *ech* in recombinant *E. coli*. Lane M, Protein weight marker (Fermentas Canada Inc., Burlington, Canada); lane 1, cell extracts of *E. coli* BL21 (pETDuet-1); lane 2, cell extracts of *E. coli* BL21 (pETDuet-*ech*-*fcs*). Marker sizes are indicated on the left in kilodaltons. The two arrows indicate the over-expressed target proteins of different sizes, the smaller one corresponds to Ech (∼32 kDa), and the larger one corresponds to Fcs (∼52 kDa).

The *fcs* and *ech* genes were subcloned into the two multiple cloning sites (MCSI and MCSII) of pETDuet-1 to obtain pETDuet-*ech*-*fcs*, and then it was introduced into *E. coli* BL21 (DE3) for co-expression. It was clear from the SDS-PAGE analysis that both genes were successfully expressed in *E. coli*. Two extra protein bands of approximately 52 and 32 kDa were observed in the extracts of the IPTG-induced recombinant *E. coli* BL21 (pETDuet-*ech*-*fcs*) cells; whereas no activity or enhanced bands were detected in the control where the expression vector contained no insert (Figure 1).

**Figure 2:**
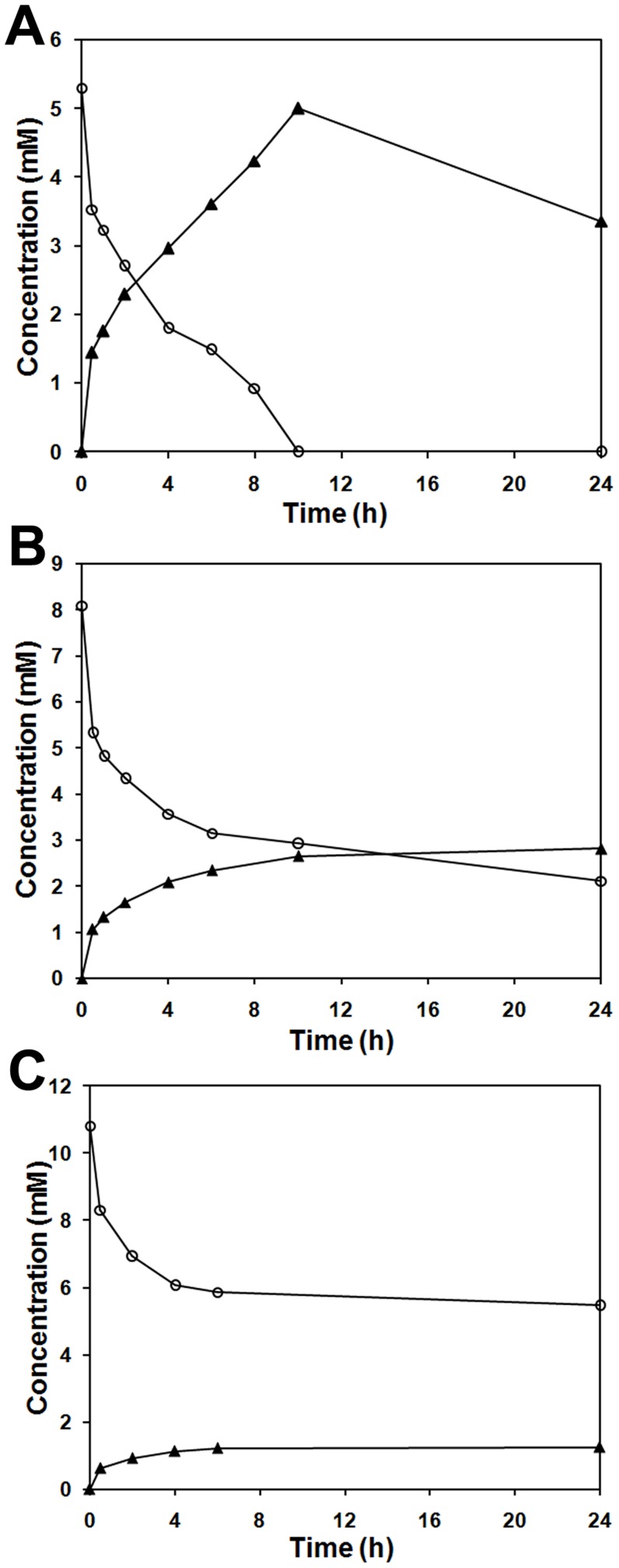
Biotransformation of ferulic acid to vanillin by resting cells of recombinant *E. coli* BL21 (pETDuet-*ech*-*fcs*). The concentrations of ferulic acid in the reaction mixture were 5.3 mM (**A**), 8.1 mM (**B**), and 10.8 mM (**C**), respectively. Symbols: ○, ferulic acid; ▴, vanillin.

**Figure 3:**
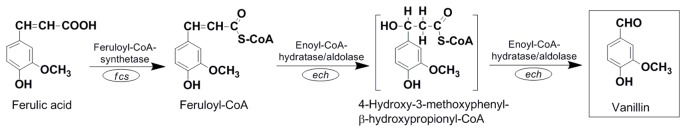
The proposed pathway of vanillin synthesis from ferulic acid in *Streptomyces* sp. strain V-1. The compound in square bracket is not detected directly.

With the IPTG-induced *E. coli* BL21 (pETDuet-*ech*-*fcs*) cells, the resting cell biotransformation was carried out with 3 different concentrations of ferulic acid, which were 5.3, 8.1 and 10.8 mM, respectively (Figure 2). After 10-h biotransformation, the highest final molar conversion of 94.3% (5.3 mM ferulic acid to 5.0 mM vanillin) was readily obtained. The other two molar conversions after 24-h transformation were only 34.6% (8.1 mM ferulic acid to 2.8 mM vanillin) and 12.0% (10.8 mM ferulic acid to 1.3 mM vanillin), respectively. The quantitative detection analysis using GC showed that co-expression of these two genes *fcs* and *ech* enabled *E. coli* to convert ferulic acid to vanillin (Figure 2), whereas the *E. coli* BL21 (pETDuet-1) cells with no insert were unable to produce vanillin from ferulic acid (data not shown). Thus, we could conclude from these results that the *fcs* and *ech* genes, encoding Fcs and Ech, respectively, were responsible for vanillin production from ferulic acid in *Streptomyces* sp. strain V-1. All of the data suggested that the pathway of vanillin synthesis from ferulic acid in *Streptomyces* sp. strain V-1 was also a coenzyme A-dependent, non-*β*-oxidative pathway (Figure 3), which was first reported by Gasson [13].

### Purification of the Recombinant Fcs and Ech

**Figure 4:**
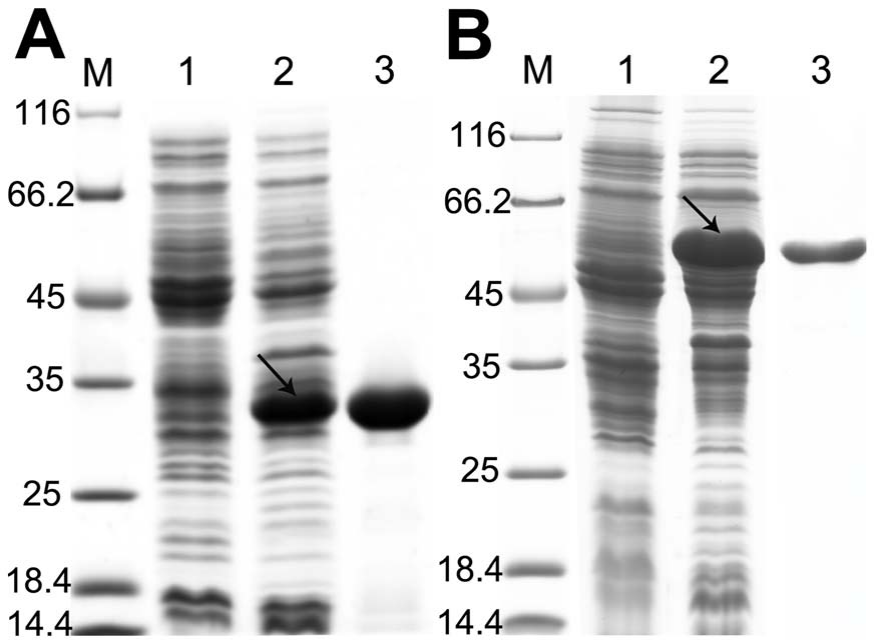
Purification of his_6_-tagged Ech and Fcs. **A:** SDS-PAGE of purified protein Ech. Lane M, Protein weight marker (Fermentas); lane 1, cell extracts of *E. coli* (pET-28a); lane 2, cell extracts of *E. coli* (pET28a-*ech*); lane 3, purified Ech (∼32 kDa). **B:** SDS-PAGE of purified protein Fcs. Lane M, Protein weight marker (Fermentas); lane 1, cell extracts of *E. coli* (pET-28a); lane 2, cell extracts of *E. coli* (pET28a-*fcs*); lane 3, purified Fcs (∼52 kDa). Marker sizes are indicated on the left in kilodaltons. The arrow indicates the over-expressed target protein.

Protein bands of ∼52-kDa and ∼32-kDa were clearly observed in the extracts of the recombinant strain *E. coli* BL21 (DE3) harboring pET28a-*fcs* and pET28a-*ech*, respectively, whereas no enhanced 52-kDa or 32-kDa protein band was observed in the control without the insert fragment (Figure 4A and 4B). The size of the target protein was in good agreement with the deduced molecular mass of expressed Fcs (51.9 kDa) and Ech (31.9 kDa).

His_6_-tagged Fcs was purified with Ni-NTA affinity column chromatography followed by anion exchange chromatography under denaturing conditions (Table 3). The protein seemed to be >95% pure, as determined from the presence of a single band (52 kDa) on the gel (Figure 4B). His_6_-tagged Ech was purified only using the Ni-NTA affinity column, and then it was desalted for further use. The purity of Ech was also more than 95%, as observed from SDS-PAGE analysis (Figure 4A).

**Table 3:** Purification of Fcs isolated from the recombinant strain *E. coli* (pET28a-*fcs*).

Step	Totalvolume (mL)	Totalprotein (mg)	Totalactivity (U)	Specific activity (U·mg^−1^)	Yield (%)	Purification factor (Fold)
Crude extract	53	227.9	2217.5	9.73	100	1.0
Ni-NTA affinity	15.4	43.7	1933.3	44.2	87.2	4.5
Anion exchange (Hitrap Q Sepharose XL)	9.6	3.74	264.0	70.6	11.9	7.3

### Characterization of the Purified Fcs

**Figure 5:**
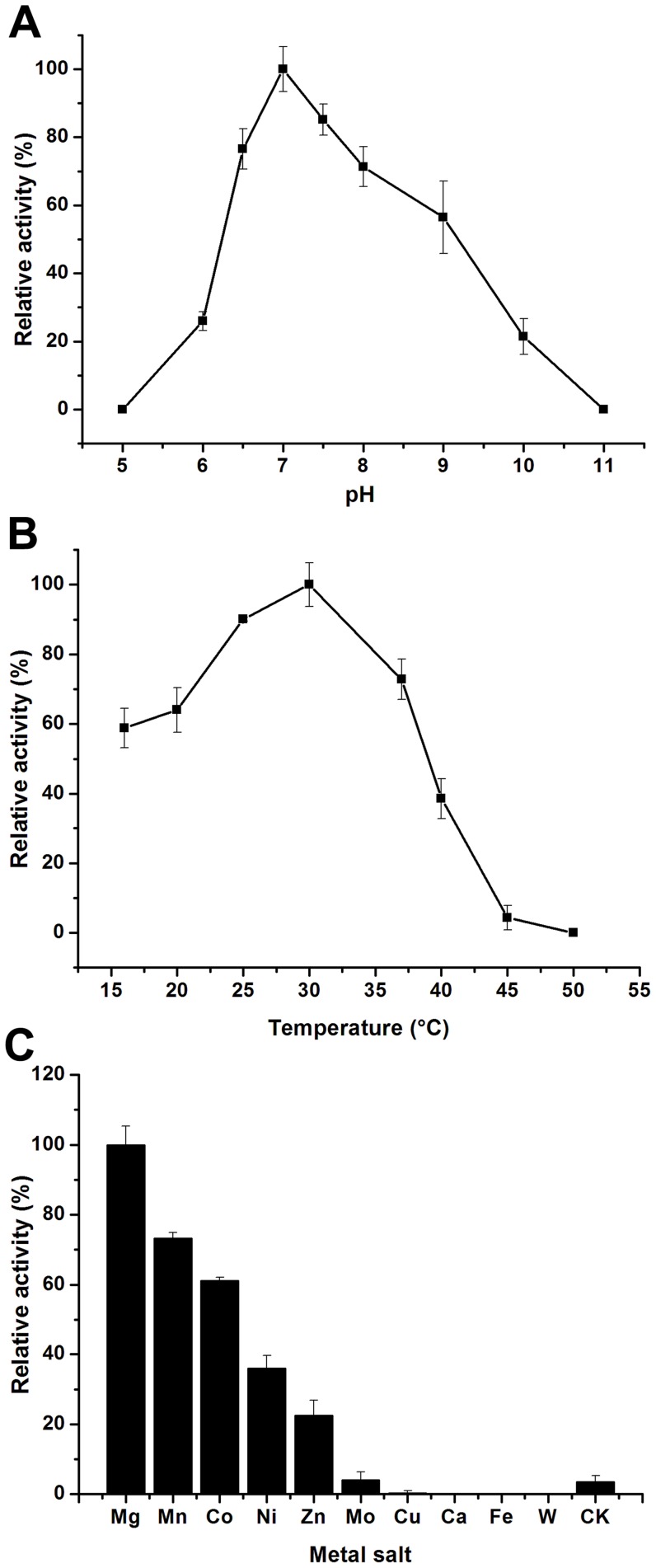
Characterization of Fcs. **A:** Effects of pH on the enzymatic activity of Fcs. **B:** Effects of temperatures on the enzymatic activity of Fcs. **C:** Effects of metal salts on the enzymatic activity of Fcs. Values represent the means of three independent experiments; the error bars represent standard deviations.

We investigated the enzymatic activity of purified Fcs for the transformation of ferulic acid to feruloyl-CoA. The enzyme Fcs displayed maximal activity at pH 7.0 in 100 mM potassium phosphate buffer. At pH values ≤5.0 or ≥11.0, a substantial loss of the activity was noted (Figure 5A). The optimal temperature for enzymatic catalysis was 30°C. High temperature (≥45°C) resulted in protein denaturation, while low temperature (16°C) seemed to have little effect on the enzyme activity (Figure 5B). The specific activity of the enzyme Fcs under optimal conditions (30°C and pH 7.0) was approximately 54.0 U mg^−1^. To assess the effects of metal salts on the Fcs activity, 10 kinds of metal salts (Mg^2+^, Mn^2+^, Co^2+^, Ni^2+^, Zn^2+^, Cu^2+^, Ca^2+^, Fe^2+^, MoO_4_^2−^, and WO_4_^2−^) were applied at a final concentration of 2.5 mM in the reaction mixture. If the specific activity obtained with 2.5 mM Mg^2+^ was defined as 100%, replacement of Mg^2+^ in the reaction mixture with 2.5 mM Mn^2+^, Co^2+^, Ni^2+^, Zn^2+^, or MoO_4_^2−^ exhibited 73.3%, 61.2%, 36.0%, 22.6%, or 4.1% of the activity, respectively (Figure 5C). However, Cu^2+^, Ca^2+^, Fe^2+^, and WO_4_^2−^ almost completely abolished the enzymatic activity of Fcs (Figure 5C). Protein precipitation was observed in aqueous solution when the metal salt was replaced by Cu^2+^, Ca^2+^, Fe^2+^, or WO_4_^2−^ in the mixture. Possibly, Fcs inactivation may be the result of protein denaturation owing to the addition of Ca^2+^ and another three kinds of heavy metal salts (Cu^2+^, Fe^2+^, and WO_4_^2−^).

The kinetic constants of Fcs were determined by changing the concentration of ferulic acid from 0.175 mM to 0.7 mM. Recombinant Fcs displayed standard Michaelis-Menten kinetics, and the *K*_m_ and *V*_max_ values were calculated from the Lineweaver-Burk double reciprocal plot (Figure S5) as 0.35 mM and 78.2 U mg^−1^ for ferulic acid, respectively. The *k*_cat_ value for Fcs was 67.7 s^−1^. Therefore, the catalytic efficiency (*k*_cat_/*K*_m_) value of Fcs was 193.4 mM^−1^ s^−1^ for ferulic acid.

### Substrate Specificity of Fcs

To determine the substrate specificity of Fcs, 6 different cinnamic acid derivatives were applied. Each compound was incubated at 0.5 mM with 30.6 µg purified protein Fcs in the 1-mL reaction mixture which contained 100 mM potassium phosphate buffer (pH 7.0), 2.5 mM MgCl_2_, 2 mM ATP, and 0.4 mM CoA. The enzymatic reaction was stopped by the addition of 6 M HCl after the 3-min incubation at 30°C. Then the sample was extracted with butyl acetate for GC analysis. The highest activity of Fcs was obtained when dealing with ferulic acid as the substrate (Table 4). Also, Fcs could transform caffeic acid and *p*-coumaric acid at a similar rate, and *trans*-cinnamic acid was transformed at about half the rate of caffeic acid transformation (Table 4). However, Fcs was unable to transform 3-methoxycinnamic acid and 4-methoxycinnamic acid.

**Table 4:** Substrate specificity of Fcs.

Substrate	Transformation activity ± SD a (µmol/min·mg)	Transformation activity ± SD [^11^] (nmol/min·mg)
Ferulic acid	2.77±0.16	120±14
Caffeic acid	1.90±0.05	99±17
*p*-Coumaric acid	1.88±0.09	110±23
*trans*-Cinnamic acid	1.03±0.10	0
3-Methoxycinnamic acid	0	0
4-Methoxycinnamic acid	0	58±6

a The transformation activity of Fcs was determined in this work.

### Activity Assay of the Purified Ech

Ech has been proposed to be involved in the biotransformation of feruloyl-CoA to vanillin. Overhage *et al*. used 4-hydroxy-3-methoxyphenyl-β-hydroxypropionyl-CoA (HMPHP-CoA) as the substrate for Ech enzyme assay, and its conversion to vanillin was confirmed by HPLC analysis [7]. Due to the unavailable HMPHP-CoA, it was unable to perform the enzymatic assay of purified Ech with HMPHP-CoA as the reaction substrate. Alternatively, we carried out the enzymatic reaction in a 2-mL mixture of 100 mM potassium phosphate buffer (pH 7.0), 2.5 mM MgCl_2_, 0.7 mM ferulic acid, 2 mM ATP, 0.4 mM CoA, and an appropriate amount of the purified proteins Fcs (of which the activity was already assayed above) and Ech. The reaction was activated by the addition of ATP. At regular time intervals, the reaction was stopped and 2-mL samples were obtained for GC analysis. Both the degradation of ferulic acid, catalyzed by Fcs, and production of vanillin, catalyzed by Ech, were observed and analyzed qualitatively from the GC spectrum (Figure S4). Therefore, the enoyl-CoA hydratase/aldolase of *Streptomyces* sp. strain V-1 was thought to play an important role in the biosynthesis pathway of vanillin from ferulic acid.

## Discussion

Using genomic DNA of *Streptomyces* sp. strain V-1 as a template and based on sequence information obtained from several genes responsible for vanillin synthesis from ferulic acid from various organisms reported previously, two critical genes (*fcs* and *ech*) with the same functions from strain V-1 were isolated. The relative molecular masses of Fcs and Ech calculated by the amino acid (aa) sequences were approximately 51.9 kDa and 32.0 kDa, respectively. The deduced amino acid sequences of *fcs* of strain V-1 showed 100% aa identity to Fcs from the gram-positive bacterium *Amycolatopsis* sp. strain HR167 [10] and 99.8% aa identity to that of *Amycolatopsis* sp. strain ATCC 39116 [8], but no significant similarity to *Pseudomonas* [7] (Figure S2A and Table S1). For the gene product of *ech*, the highest sequence similarity was found to correspond to Ech from *Amycolatopsis* sp. ATCC 39116 (100% identity in a 287-aa overlap) and *Amycolatopsis* sp. HR167 (99.0% identity in a 284-aa overlap) [8], [10]. However, it exhibited only a 51.6% aa identity to Ech from *Pseudomonas* sp. strain HR199 [7] (Figure S3A and Table S1). According to the alignment results above and the constructed phylogenetic tree (Figure S2B and Figure S3B), *Streptomyces* sp. V-1 was considered to have the nearest phylogenetic relationship with *Amycolatopsis* sp. strain HR167 and *Amycolatopsis* sp. strain ATCC 39116. Interestingly, both *Streptomyces* and *Amycolatopsis* strains were reported to be the most promising strains for industrial vanillin production (>10 g L^−1^) [9], [14], [15]. This result might suggest that the genes, proteins, or regulatory mechanisms involved in vanillin production in these strains may have some unique features, enabling these strains to efficiently produce vanillin in elevated quantities.

Although the sequences encoding putative feruloyl-CoA synthetase/ligase and enoyl-CoA hydratase/aldolase were identified by sequence homology in several *Streptomyces* annotated genomes, such as *Streptomyces hygroscopicus* subsp. jinggangensis 5008 [23], *Streptomyces* sp. SCC 2136 [24], and *Streptomyces* sp. e14 (unpublished data), further studies on heterologous expression, transformation ability identification, and enzyme activity determination, were not performed so far. The genes *fcs* and *ech* from *Streptomyces* sp. strain V-1 were heterologously co-expressed in *E. coli* and resting cells of the recombinant strain were prepared for the biotransformation experiment of ferulic acid at 3 different final concentrations (5.3, 8.1 and 10.8 mM). When 5.3 mM ferulic acid was applied, the highest final molar conversion was found to be 94.3% (5.3 mM ferulic acid to 5.0 mM vanillin) after 10-h transformation. Besides that, we obtained the molar conversions of 34.6% for 8.1 mM ferulic acid to 2.8 mM vanillin and only 12.0% for 10.8 mM ferulic acid to 1.3 mM vanillin, respectively, after an incubation of 24 h, when there were still a large quantity of substrates remaining in the reaction mixture. Both the vanillin production and the conversion rate of ferulic acid were decreased by the increasing initial concentration of ferulic acid, which might result from the substrate inhibition effect. Achterholt *et al.* [10] reported that 2.3 mM vanillin was obtained from 5.15 mM ferulic acid after 6-h incubation and a maximum of 3.1 mM vanillin after 23 h, through the biotransformation of ferulic acid to vanillin by resting cells of the recombinant *E. coli* strain. Overhage *et al.* [7] obtained the maximum of about 1.9 mM vanillin from 3.7 mM ferulic acid after 2-h incubation with resting cells of their constructed recombinant *E. coli* strain. In the field of bioengineering, as a non-native vanillin producer, *E. coli* was transformed with the genes responsible for vanillin synthesis to produce vanillin with its well-developed fermentation system. And Lee *et al.* [25] reported the highest vanillin production from ferulic acid (33.8 mM vanillin in 24 h, and molar conversion yield of 86.6%) using recombinant *E. coli*.

Following the isolation of the *ech* and *fcs* genes from strain V-1 and their functional confirmation, biochemical characterizations of the two proteins Ech and Fcs were performed in detail. We studied the optimal pH, temperature, and the metal ion dependency for Fcs. Also, the kinetic properties of Fcs were assayed with the apparent *K*_m_, *k*_cat_, and *V*_max_ values to be 0.35 mM, 67.7 s^−1^, and 78.2 U mg^−1^, respectively. And the catalytic efficiency (*k*_cat_/*K*_m_) value of Fcs was 193.4 mM^−1^ s^−1^ for ferulic acid. The feruloyl-CoA synthetase from *Streptomyces* sp. strain V-1 was responsible for the transformation of ferulic acid to the intermediate feruloyl-CoA. In the strain *Streptomyces coelicolor* A3(2), a 4-coumarate:coenzyme A ligase (4CL) was found to have more than 40% identity in amino acid sequence to plant 4CLs, and then it was purified from recombinant *E. coli* and determined for its activity for different cinnamate derivatives [26]. Kaneko *et al.* [26] reported that the wild-type 4CL in *S. coelicolor* A3(2) showed no activity toward ferulic acid, but only one of the mutant proteins (A294G/A318G) showed the *K*_m_, *k*_cat_, and *k*_cat_/*K*_m_ values to be 0.242 mM, 0.106 s^−1^, and 0.438 mM^−1^ s^−1^, respectively. As for the plant 4CL, it played a key role in the phenylpropanoid metabolism, converting 4-coumaric acid and other hydroxycinnamates into the corresponding CoA thiol esters which were precursors for many important plant secondary metabolites, such as flavonoids, lignin, and coumarins [27], [28]. Ehlting *et al.* [27] reported on the cloning and expression studies of the 3 members of 4CL, At4CL1, At4CL2, and At4CL3, from *Arabidopsis thaliana*, and they determined the kinetic constant *K*_m_ of At4CL1 and At4CL3 for ferulic acid (while At4CL2 was not capable of converting ferulic acid) to be 0.199 mM (for At4CL1) and 0.166 mM (for At4CL3), respectively. Although both the plant 4CLs [27], [28] and the 4CL-like enzyme characterized from *S. coelicolor* A3(2) [26] could convert ferulic acid to the CoA thiol ester feruloyl-CoA, these enzymes and the corresponding products were mostly intended for the synthesis of various phenylpropanoid-derived compounds for the plant secondary metabolism instead of the production of the flavoring agent vanillin. Therefore, the detailed biochemical characterizations of Fcs from *Streptomyces* sp. strain V-1 in this study may provide more practical information at the enzymatic level for further research in the biotransformation process of natural vanillin from ferulic acid.

In addition, we investigated the substrate specificity of the purified feruloyl-CoA synthetase from *Streptomyces* sp. strain V-1 for several cinnamic acid derivatives (Table 4), which was previously studied by Masai *et al.* [11] with the cell extracts of recombinant *E. coli* carrying the 3 vanillin production genes (*ferA*, *ferB*, and *ferB2*) of *Sphingomonas paucimobilis* strain SYK-6. Fcs from strain V-1 showed obvious activity to ferulic acid, caffeic acid, *p*-coumaric acid, and *trans*-cinnamic acid which were also examined in *S. paucimobilis* strain SYK-6 [11]. Whereas, 3-methoxycinnamic acid and 4-methoxycinnamic acid did not serve as the substrate of Fcs. Thus, the broad substrate spectra and the relatively high enzyme activity of Fcs from strain V-1 may have the potential of being used in the biotransformation process of different cinnamic acid derivatives.

## Supporting Information

Figure S1 **Alignment of the deduced amino acid sequences of** ***ech*** **from different strains.** The amino acid sequences of 5 hydratases of similar function from 5 different strains were aligned with the clustalX software. (*i*) The amino acid sequence of enoyl-CoA hydratase from *Pseudomonas* sp. HR199 [7]; (*ii*) The amino acid sequence of *p*-hydroxycinnamoyl CoA hydratase/lyase from *Pseudomonas fluorescens* AN103 [13]; (*iii*) The amino acid sequence of ferulic acid hydratase from *Pseudomonas putida* WCS358 [18]; (*iv*) The amino acid sequence of enoyl-CoA hydratase/aldolase from *Amycolatopsis* sp. HR167 [10]; (*v*) The amino acid sequence of feruloyl-CoA hydratase/lyase from *Sphingomonas paucimobilis* SYK-6 [11]. The amino acids in the frame were consistent with the degenerate primer pair P1 and P2. Amino acids were specified by standard one-letter abbreviations. *Dashes* indicate gaps introduced into the sequences to improve the alignment.
(PDF)

Figure S2 **Homologies of feruloyl-CoA synthetase from** ***Streptomyces*** **sp. V-1 with the CoA ligases from various organisms. A.** The amino acid sequence of feruloyl-CoA synthetase from *Streptomyces* sp. V-1 deduced from *fcs* (*iii*) was aligned to: the amino acid sequence of (*i*) the feruloyl-CoA synthetase from *Streptomyces hygroscopicus* subsp. jinggangensis 5008 [23], (*ii*) the feruloyl-CoA synthetase from *Streptomyces* sp. SCC 2136 [24], (*iv*) the feruloyl-CoA synthetase from *Amycolatopsis* sp. HR167 [10], (*v*) the acyl-CoA synthetase (AMP-forming)/AMP-acid ligase II from *Amycolatopsis* sp. ATCC 39116 [8], (*vi*) the O-succinylbenzoate-CoA ligase from *Streptomyces* sp. e14 (unpublished data), and (*vii*) the feruloyl-CoA synthetase from *Pseudomonas* sp. HR199 [7]. Amino acids are specified by standard one-letter abbreviations. *Dashes* indicate gaps introduced into the sequences to improve the alignment. **B.** The relationship between the feruloyl-CoA synthetase of *Streptomyces* sp. V-1 and the proteins in panel **A** was displayed as a phylogenetic tree, which was constructed on the basis of the Neighbor-Joining (NJ) method using the ClustalX and MEGA 5.0 softwares. The bar indicates 10% difference in amino acid sequence. The number at the branch point represents the percentage of 1,000 bootstrap repetitions.
(PDF)

Figure S3 **Homologies of enoyl-CoA hydratase/aldolase from** ***Streptomyces*** **sp. V-1 with the CoA hydratases/aldolases from different organisms. A.** The amino acid sequence of enoyl-CoA hydratase/aldolase from *Streptomyces* sp. V-1 deduced from *ech* gene (*i*) was aligned to: the amino acid sequence of (*ii*) the *p*-hydroxycinnamoyl CoA hydratase/lyase from *Amycolatopsis* sp. ATCC 39116 [8], (*iii*) the enoyl-CoA hydratase/aldolase from *Amycolatopsis* sp. HR167 [10], (*iv*) the *p*-hydroxycinnamoyl CoA hydratase/lyase from *Pseudonocardia* sp. P1 (unpublished data), (*v*) the 2-ketocyclohexanecarboxyl-CoA hydratase from *Rhodococcus opacus* PD630 [29], (*vi*) the *p*-hydroxycinnamoyl CoA hydratase/lyase from *Pseudomonas fluorescens* AN103 [13], and (*vii*) the enoyl-CoA hydratase from *Pseudomonas* sp. HR199 [7]. Amino acids are specified by standard one-letter abbreviations. *Dashes* indicate gaps introduced into the sequences to improve the alignment. **B.** The relationship between the enoyl-CoA hydratase/aldolase from *Streptomyces* sp. V-1 and the proteins in panel **A** was displayed as a phylogenetic tree, which was constructed on the basis of the Neighbor-Joining (NJ) method using the ClustalX and MEGA 5.0 softwares. The bar indicates 10% difference in amino acid sequence. The number at the branch point represents the percentage of 1,000 bootstrap repetitions.
(PDF)

Figure S4 **GC spectrum of the conversion from ferulic acid to vanillin by both Fcs and Ech.** The three lines indicate samples obtained at 0 min (blue), 5 min (red), and 10 min (green) during the enzymatic reactions with two purified proteins Fcs and Ech in the reaction mixture.
(PDF)

Figure S5 **Determination of the kinetic constants of Fcs.** Michaelis-Menten and Lineweaver-Burk reciprocal plots of Fcs were determined by changing the concentration of the substrate (ferulic acid) from 0.175 mM to 0.7 mM. Values represent the means of three independent experiments; the error bars represent standard deviations.
(PDF)

Table S1 **The amino acid sequence similarities of Fcs and Ech in different organisms.**
(DOC)
