# Supplementary material for: Characterization of Two Streptomyces Enzymes That Convert Ferulic Acid to Vanillin
Source: PLoS One. 2013 Jun 28;8(6):e67339. doi: 10.1371/journal.pone.0067339 (PMC3696112; doi:10.1371/journal.pone.0067339)
Supplement: Figure S1 — Alignment of the deduced amino acid sequences of ech from different strains. The amino acid sequences of 5 hydratases of similar function from 5 different strains were aligned with the clustalX software. (i) The amino acid sequence of enoyl-CoA hydratase from Pseudomonas sp. HR199 [7]; (ii) The amino acid sequence of p-hydroxycinnamoyl CoA hydratase/lyase from Pseudomonas fluorescens AN103 [13]; (iii) The amino acid sequence of ferulic acid hydratase from Pseudomonas putida WCS358 [18]; (iv) The amino acid sequence of enoyl-CoA hydratase/aldolase from Amycolatopsis sp. HR167 [10]; (v) The amino acid sequence of feruloyl-CoA hydratase/lyase from Sphingomonas paucimobilis SYK-6 [11]. The amino acids in the frame were consistent with the degenerate primer pair P1 and P2. Amino acids were specified by standard one-letter abbreviations. Dashes indicate gaps introduced into the sequences to improve the alignment. (PDF) [file pone.0067339.s001.pdf]

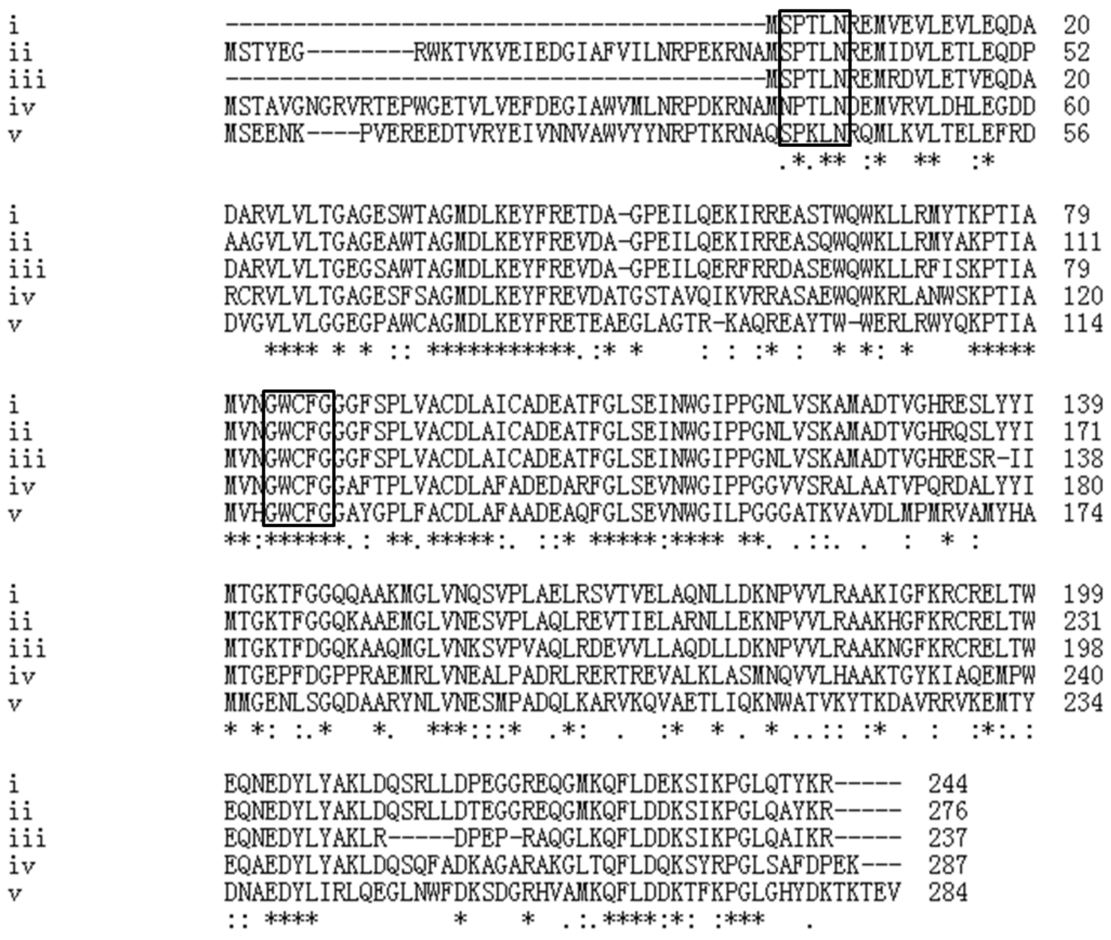

Figure S1. Alignment of the deduced amino acid sequences of *ech* from different strains. The amino acid sequences of 5 hydratases of similar function from 5 different strains were aligned with the clustalX software. (*i*) The amino acid sequence of enoyl-CoA hydratase from *Pseudomonas* sp. HR199 [7]; (*ii*) The amino acid sequence of *p*-hydroxycinnamoyl CoA hydratase/lyase from *Pseudomonas fluorescens* AN103 [13]; (*iii*) The amino acid sequence of ferulic acid hydratase from *Pseudomonas putida* WCS358 [18]; (*iv*) The amino acid sequence of enoyl-CoA hydratase/aldolase from *Amycolatopsis* sp. HR167 [10]; (*v*) The amino acid sequence of feruloyl-CoA hydratase/lyase from *Sphingomonas paucimobilis* SYK-6 [11]. The amino acids in the frame were consistent with the degenerate primer pair P1 and P2. Amino acids were

specified by standard one-letter abbreviations. *Dashes* indicate gaps introduced into the sequences to improve the alignment.
