# Supplementary material for: Characterization of Two Streptomyces Enzymes That Convert Ferulic Acid to Vanillin
Source: PLoS One. 2013 Jun 28;8(6):e67339. doi: 10.1371/journal.pone.0067339 (PMC3696112; doi:10.1371/journal.pone.0067339)
Supplement: Figure S2 — Homologies of feruloyl-CoA synthetase from Streptomyces sp. V-1 with the CoA ligases from various organisms. A. The amino acid sequence of feruloyl-CoA synthetase from Streptomyces sp. V-1 deduced from fcs (iii) was aligned to: the amino acid sequence of (i) the feruloyl-CoA synthetase from Streptomyces hygroscopicus subsp. jinggangensis 5008 [23], (ii) the feruloyl-CoA synthetase from Streptomyces sp. SCC 2136 [24], (iv) the feruloyl-CoA synthetase from Amycolatopsis sp. HR167 [10], (v) the acyl-CoA synthetase (AMP-forming)/AMP-acid ligase II from Amycolatopsis sp. ATCC 39116 [8], (vi) the O-succinylbenzoate-CoA ligase from Streptomyces sp. e14 (unpublished data), and (vii) the feruloyl-CoA synthetase from Pseudomonas sp. HR199 [7]. Amino acids are specified by standard one-letter abbreviations. Dashes indicate gaps introduced into the sequences to improve the alignment. B. The relationship between the feruloyl-CoA synthetase of Streptomyces sp. V-1 and the proteins in panel A was displayed as a phylogenetic tree, which was constructed on the basis of the Neighbor-Joining (NJ) method using the ClustalX and MEGA 5.0 softwares. The bar indicates 10% difference in amino acid sequence. The number at the branch point represents the percentage of 1,000 bootstrap repetitions. (PDF) [file pone.0067339.s002.pdf]

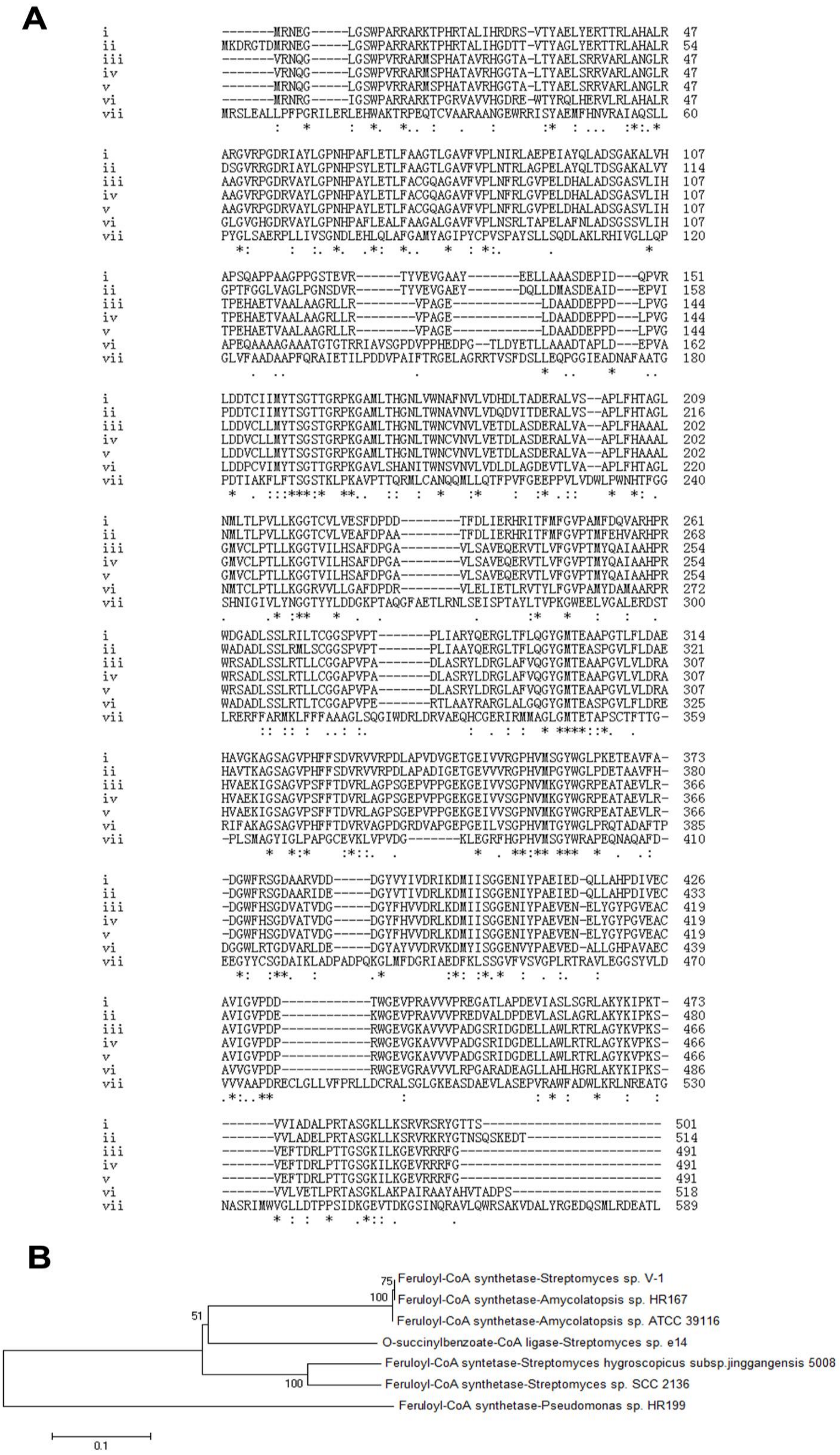
A
i -------MRNEG-----LGSWPARRARKTPHRTALIHRDRS-VTYAELYERTTRLAHALR 47
ii MKDRGTDMRNEG-----LGSWPARRARKTPHRTALIHGDTT-VTYAGLYERTTRLAHALR 54
iii -------VRNQG-----LGSWPVRRARMSPHATAVRHGGTA-LTYAELSRRVARLANGLR 47
iv -------VRNQG-----LGSWPVRRARMSPHATAVRHGGTA-LTYAELSRRVARLANGLR 47
v -------MRNQG-----LGSWPVRRARMSPHATAVRHGGTA-LTYAELSRRVARLANGLR 47
vi -------MRNRG-----IGSWPARRARKTPGRVAVVHGDRE-WTYRQLHERVLRLAHALR 47
vii MRSLEALLPFPGRILERLEHWAKTRPEQTCVAARAANGEWRRISYAEMFHNVRAIAQSLL 60
i ARGVRPGDRIAYLGPNHPAFLETLFAAGTLGAVFVPLNIRLAEPEIAYQLADSGAKALVH 107
ii DSGVRRGDRIAYLGPNHPSYLETLFAAGTLGAVFVPLNTRLAGPELAYQLTDSGAKALVY 114
iii AAGVRPGDRVAYLGPNHPAYLETLFACGQAGAVFVPLNFRLGVPELDHALADSGASVLIH 107
iv AAGVRPGDRVAYLGPNHPAYLETLFACGQAGAVFVPLNFRLGVPELDHALADSGASVLIH 107
v AAGVRPGDRVAYLGPNHPAYLETLFACGQAGAVFVPLNFRLGVPELDHALADSGASVLIH 107
vi GLGVGHGDRVAYLGPNHPAFLEALFAAGALGAVFVPLNSRLTAPELAFNLADSGSSVLIH 107
vii PYGLSAERPLLIVSGNDLEHLQLAFGAMYAGIPYCPVSPAYSLLSQDLAKLRHIVGLLQP 120
i APSQAPPAAGPPGSTEVR------TYVEVGAAY-------EELLAAASDEPID---QPVR 151
ii GPTFGGLVAGLPGNSDVR------TYVEVGAEY-------DQLLDMASDEAID---EPVI 158
iii TPEHAETVAALAAGRLLR--------VPAGE------------LDAADDEPPD---LPVG 144
iv TPEHAETVAALAAGRLLR--------VPAGE------------LDAADDEPPD---LPVG 144
v TPEHAETVAALAAGRLLR--------VPAGE------------LDAADDEPPD---LPVG 144
vi APEQAAAAGAAATGTGTRRIAVSGPDVPPHEDPG--TLDYETLLAAADTAPLD---EPVA 162
vii GLVFAADAAPFQRAIETILPDDVPAIFTRGELAGRRTVSFDSLLEQPGGIEADNAFAATG 180
i LDDTCIIMYTSGTTGRPKGAMLTHGNLVWNAFNVLVDHDLTADERALVS--APLFHTAGL 209
ii PDDTCIIMYTSGTTGRPKGAMLTHGNLTWNAVNVLVDQDVITDERALVS--APLFHTAGL 216
iii LDDVCLLMYTSGSTGRPKGAMLTHGNLTWNCVNVLVETDLASDERALVA--APLFHAAAL 202
iv LDDVCLLMYTSGSTGRPKGAMLTHGNLTWNCVNVLVETDLASDERALVA--APLFHAAAL 202
v LDDVCLLMYTSGSTGRPKGAMLTHGNLTWNCVNVLVETDLASDERALVA--APLFHAAAL 202
vi LDDPCVIMYTSGTTGRPKGAVLSHANITWNSVNVLVDLDLAGDEVTLVA--APLFHTAGL 220
vii PDTIAKFLFTSGSTKLPKAVPTTQRMLCANQQMLLQTFPVFGEEPPVLVDWLPWNHTFGG 240
i NMLTLPVLLKGGTCVLVESFDPDD--------TFDLIERHRITFMFGVPAMFDQVARHPR 261
ii NMLTLPVLLKGGTCVLVEAFDPAA--------TFDLIERHRITFMFGVPTMFEHVARHPR 268
iii GMVCLPTLLKGGTVILHSAFDPGA--------VLSAVEQERVTLVFGVPTMYQAIAAHPR 254
iv GMVCLPTLLKGGTVILHSAFDPGA--------VLSAVEQERVTLVFGVPTMYQAIAAHPR 254
v GMVCLPTLLKGGTVILHSAFDPGA--------VLSAVEQERVTLVFGVPTMYQAIAAHPR 254
vi NMTCLPTLLKGGRVVLLGAFDPDR--------VLELIETLRVTYLFGVPAMYDAMAARPR 272
vii SHNIGIVLYNGGTYYLDDGKPTAQGFAETLRNLSEISPTAYLTVPKGWEELVGALERDST 300
i WDGADLSSLRILTCGGSPVPT-------PLIARYQERGLTFLQGYGMTEAAPGTLFLDAE 314
ii WADADLSSLRMLSCGGSPVPT-------PLIAAYQERGLTFLQGYGMTEASPGVLFLDAE 321
iii WRSADLSSLRTLLCGGAPVPA-------DLASRYLDRGLAFVQGYGMTEAAPGVLVLDRA 307
iv WRSADLSSLRTLLCGGAPVPA-------DLASRYLDRGLAFVQGYGMTEAAPGVLVLDRA 307
v WRSADLSSLRTLLCGGAPVPA-------DLASRYLDRGLAFVQGYGMTEAAPGVLVLDRA 307
vi WADADLSSLRTLTCGGAPVPE-------RTLAAYRARGLALGQGYGMTEASPGVLFLDRE 325
vii LRERFFARMKLFFFAAAGLSQGIWDRLDRVAEQHCGERIRMMAGLGMTETAPSCTFTTG- 359
i HAVGKAGSAGVPHFFSDVRVVRPDLAPVDVGETGEIVVRGPHVMSGYWGLPKETEAVFA- 373
ii HAVTKAGSAGVPHFFSDVRVVRPDLAPADIGETGEVVVRGPHVMPGYWGLPDETAAVFH- 380
iii HVAEKIGSAGVPSFFTDVRLAGPSGEPVPPGEKGEIVVSGPNVMKGYWGRPEATAEVLR- 366
iv HVAEKIGSAGVPSFFTDVRLAGPSGEPVPPGEKGEIVVSGPNVMKGYWGRPEATAEVLR- 366
v HVAEKIGSAGVPSFFTDVRLAGPSGEPVPPGEKGEIVVSGPNVMKGYWGRPEATAEVLR- 366
vi RIFAKAGSAGVPHFFTDVRVAGPDGRDVAPGEPGEILVSGPHVMTGYWGLPRQTADAFTP 385
vii -PLSMAGYIGLPAPGCEVKLVPVDG-------KLEGRFHGPHVMSGYWRAPEQNAQAFD- 410
i -DGWFRSGDAARVDD-----DGYVYIVDRIKDMIISGGENIYPAEIED-QLLAHPDIVEC 426
ii -DGWFRSGDAARIDE-----DGYVTIVDRLKDMIISGGENIYPAEIED-QLLAHPDIVEC 433
iii -DGWFHSGDVATVDG-----DGYFHVVDRLKDMIISGGENIYPAEVEN-ELYGYPGVEAC 419
iv -DGWFHSGDVATVDG-----DGYFHVVDRLKDMIISGGENIYPAEVEN-ELYGYPGVEAC 419
v -DGWFHSGDVATVDG-----DGYFHVVDRLKDMIISGGENIYPAEVEN-ELYGYPGVEAC 419
vi DGGWLRTGDVARLDE-----DGYAYVVDRVKDMYISGGENVYPAEVED-ALLGHPAVAEC 439
vii EEGYYCSGDAIKLADPADPQKGLMFDGRIAEDFKLSSGVFVSVGPLRTRAVLEGGSYVLD 470
i AVIGVPDD------------TWGEVPRAVVVPREGATLAPDEVIASLSGRLAKYKIPKT- 473
ii AVIGVPDE------------KWGEVPRAVVVPREDVALDPDEVLASLAGRLAKYKIPKS- 480
iii AVIGVPDP------------RWGEVGKAVVVPADGSRIDGDELLAWLRTRLAGYKVPKS- 466
iv AVIGVPDP------------RWGEVGKAVVVPADGSRIDGDELLAWLRTRLAGYKVPKS- 466
v AVIGVPDP------------RWGEVGKAVVVPADGSRIDGDELLAWLRTRLAGYKVPKS- 466
vi AVVGVPDP------------RWGEVGRAVVVLRPGARADEAGLLAHLHGRLAKYKIPKS- 486
vii VVVAAPDRECLGLLVFPRLLDCRALSGLGKEASDAEVLASEPVRAWFADWLKRLNREATG 530
i -------VVIADALPRTASGKLLKSRVRSRYGTTS------------------------ 501
ii -------VVLADELPRTASGKLLKSRVRKRYGTNSQSKEDT------------------ 514
iii -------VEFTDRLPTTGSGKILKGEVRRRFG--------------------------- 491
iv -------VEFTDRLPTTGSGKILKGEVRRRFG--------------------------- 491
v -------VEFTDRLPTTGSGKILKGEVRRRFG--------------------------- 491
vi -------VVLVETLPRTASGKLAKPAIRAAYAHVTADPS-------------------- 518
vii NASRIMWVGLLDTPPSIDKGEVTDKGSINQRAVLQWRSAKVDALYRGEDQSMLRDEATL 589
B
75
Feruloyl-CoA synthetase-Streptomyces sp. V-1
100
Feruloyl-CoA synthetase-Amycolatopsis sp. HR167
51
Feruloyl-CoA synthetase-Amycolatopsis sp. ATCC 39116
O-succinylbenzoate-CoA ligase-Streptomyces sp. e14
Feruloyl-CoA syntetase-Streptomyces hygroscopicus subsp.jinggangensis 5008
100
Feruloyl-CoA synthetase-Streptomyces sp. SCC 2136
Feruloyl-CoA synthetase-Pseudomonas sp. HR199
0.1

Figure S2. Homologies of feruloyl-CoA synthetase from *Streptomyces* sp. V-1 with the CoA ligases from various organisms. A. The amino acid sequence of feruloyl-CoA synthetase from *Streptomyces* sp. V-1 deduced from *fcs* (*iii*) was aligned to: the amino acid sequence of (*i*) the feruloyl-CoA synthetase from *Streptomyces hygroscopicus* subsp. jinggangensis 5008 [23], (*ii*) the feruloyl-CoA synthetase from *Streptomyces* sp. SCC 2136 [24], (*iv*) the feruloyl-CoA synthetase from *Amycolatopsis* sp. HR167 [10], (*v*) the acyl-CoA synthetase (AMP-forming) /AMP-acid ligase II from *Amycolatopsis* sp. ATCC 39116 [8], (*vi*) the O-succinylbenzoate-CoA ligase from *Streptomyces* sp. e14 (unpublished data), and (*vii*) the feruloyl-CoA synthetase from *Pseudomonas* sp. HR199 [7]. Amino acids are specified by standard one-letter abbreviations. *Dashes* indicate gaps introduced into the sequences to improve the alignment. **B.** The relationship between the feruloyl-CoA synthetase of *Streptomyces* sp. V-1 and the proteins in panel **A** was displayed as a phylogenetic tree, which was constructed on the basis of the Neighbor-Joining (NJ) method using the ClustalX and MEGA 5.0 softwares. The bar indicates 10% difference in amino acid sequence. The number at the branch point represents the percentage of 1,000 bootstrap repetitions.
