# Supplementary material for: Characterization of Two Streptomyces Enzymes That Convert Ferulic Acid to Vanillin
Source: PLoS One. 2013 Jun 28;8(6):e67339. doi: 10.1371/journal.pone.0067339 (PMC3696112; doi:10.1371/journal.pone.0067339)
Supplement: Figure S3 — Homologies of enoyl-CoA hydratase/aldolase from Streptomyces sp. V-1 with the CoA hydratases/aldolases from different organisms. A. The amino acid sequence of enoyl-CoA hydratase/aldolase from Streptomyces sp. V-1 deduced from ech gene (i) was aligned to: the amino acid sequence of (ii) the p-hydroxycinnamoyl CoA hydratase/lyase from Amycolatopsis sp. ATCC 39116 [8], (iii) the enoyl-CoA hydratase/aldolase from Amycolatopsis sp. HR167 [10], (iv) the p-hydroxycinnamoyl CoA hydratase/lyase from Pseudonocardia sp. P1 (unpublished data), (v) the 2-ketocyclohexanecarboxyl-CoA hydratase from Rhodococcus opacus PD630 [29], (vi) the p-hydroxycinnamoyl CoA hydratase/lyase from Pseudomonas fluorescens AN103 [13], and (vii) the enoyl-CoA hydratase from Pseudomonas sp. HR199 [7]. Amino acids are specified by standard one-letter abbreviations. Dashes indicate gaps introduced into the sequences to improve the alignment. B. The relationship between the enoyl-CoA hydratase/aldolase from Streptomyces sp. V-1 and the proteins in panel A was displayed as a phylogenetic tree, which was constructed on the basis of the Neighbor-Joining (NJ) method using the ClustalX and MEGA 5.0 softwares. The bar indicates 10% difference in amino acid sequence. The number at the branch point represents the percentage of 1,000 bootstrap repetitions. (PDF) [file pone.0067339.s003.pdf]

A

```
i               MSTAVGNGRVRTEPWGETVLVEFDEG-IAWVMLNRPDKRNAMNPTLNDEMVRVLDHLEGD  59
ii              MSTAVGNGRVRTEPWGETVLVEFDEG-IAWVMLNRPDKRNAMNPTLNDEMVRVLDHLEGD  59
iii             MSTAVGNGRVRTEPWGETVLVEFDEG-IAWVMLNRPDKRNAMNPTLNDEMVRVLDHLEGD  59
iv              ----------MTEPWGNTVLVEFDEG-IAWVSLNRPDKRNAMSPTLNDEMVATLDALEGD  49
v               MSDNIN------EPWGNTVLVEFDEHRIAWVRFNRPDKRNAMNPTLNREMFATLDHLEDD  54
vi              MS--------TYEGRWKTVKVEIEDG-IAFVILNRPEKRNAMSPTLNREMIDVLETLEQD  51
vii             ----------------------------------------MSPTLNREMVEVLEVLEQD  19
                                                        *.**** **. .*: ** *

i               DRCRVLVLTGAGESFSAGMDLKEYFREVDATGSTAVQIKVRRASAEWQWKRLANWSKPTI  119
ii              DRCRVLVLTGAGESFSAGMDLKEYFREVDATGSTAVQIKVRRASAEWQWKRLANWSKPTI  119
iii             DRCRVLVLTGAGESFSAGMDLKEYFREVDATGSTAVQIKVRRASAEWQWKRLANWSKPTI  119
iv              PRCRVLVLTGSGDAFSAGMDLKEYFREVDESGDPGLQIRVRRASAEWQWKRLSNWSKPTV  109
v               DRCAVVVLTGSDDAFSAGMDLKEYFRETDGK-PRSVQHRVRGEGTNWQYRKLLNYAKPTI  113
vi              PAAGVLVLTGAGEAWTAGMDLKEYFREVDAG-PEILQEKIRREASQWQWKLLRMYAKPTI  110
vii             ADARVLVLTGAGESWTAGMDLKEYFRETDAG-PEILQEKIRREASTWQWKLLRMYTKPTI  78
                  . *:****:.::::***********.*      :* ::*  .: **:: *  ::***:

i               AMVNGWCFGGAFTPLVACDLAFADEDARFGLSEVNWGIPPGGVVSRALAATVPQRDALYY  179
ii              AMVNGWCFGGAFTPLVACDLAFADEDARFGLSEVNWGIPPGGVVSRALAATVPQRDALYY  179
iii             AMVNGWCFGGAFTPLVACDLAFADEDARFGLSEVNWGIPPGGVVSRALAATVPQRDALYY  179
iv              AMVNGWCFGGAFTPLVACDLAISDEEAQYGLSEINWGIPPGGVVSRALAATVSQRDALYF  169
v               AMVNGWCFGGAFVPLVSCDLAIASEDATFGLSEVNWGVTPGNLVTRALAETIPTRDAMFY  173
vi              AMVNGWCFGGGFSPLVACDLAICADEATFGLSEINWGIPPGNLVSKAMADTVGHRQSLYY  170
vii             AMVNGWCFGGGFSPLVACDLAICADEATFGLSEINWGIPPGNLVSKAMADTVGHRESLYY  138
                **********.* ***:****:.  ::* :****:***:.**.:*::*:* *:  *:::::

i               IMTGEPFDGRRAAEMRLVNEALPADRLRERTREVALKLASMNQVVLHAAKTGYKIAQEMP  239
ii              IMTGEPFDGRRAAEMRLVNEALPADRLRERTREVALKLASMNQVVLHAAKTGYKIAQEMP  239
iii             IMTGEPFDGPPRAEMRLVNEALPADRLRERTREVALKLASMNQVVLHAAKTGYKIAQEMP  239
iv              IMTGEPFDGRRAAGMRLVNEAVPAGRLRERTRELALKLASMNPVVMRAAKVGYKLAQEMP  229
v               IMTGQHFDGKRASEMRLVNEAVPADQLRARTIELALQLTSLSQWVVRGAKMGFRHSRLMS  233
vi              IMTGKTFGGQKAAEMGLVNESVPLAQLREVTIELARNLLEKNPVVLRAAKHGFKRCRELT  230
vii             IMTGKTFGGQQAAKMGLVNQSVPLAELRSVTVELAQNLLDKNPVVLRAAKIGFKRCRELT  198
                ****: *.*   : * ***:::*  .**  * *:* :* . .  *::.** *:: .: :.

i               WEQAEDYLYAKLDQSQFADKAGARAKGLTQFLDQKSYRPGLSAFDPEK-  287
ii              WEQAEDYLYAKLDQSQFADKAGARAKGLTQFLDQKSYRPGLSAFDPEK-  287
iii             WEQAEDYLYAKLDQSQFADKAGARAKGLTQFLDQKSYRPGLSAFDPEK-  287
iv              WEQAEDYLYAKLEQSQFLDAERGRQQGLSQFLDDKSYRPGLSAYASE--  276
v               WEAAEDYLYAKHDQAILFDTD-AREQGMTQFLDDKTYRPGLGTYATTAE  281
vi              WEQNEDYLYAKLDQSRLLDTEGGREQGMKQFLDDKSIKPGLQAYKR---  276
vii             WEQNEDYLYAKLDQSRLLDPEGGREQGMKQFLDEKSIKPGLQTYKR---  244
                **  ******* :*: : *   .* :*:.****:*: :*** ::
```

B

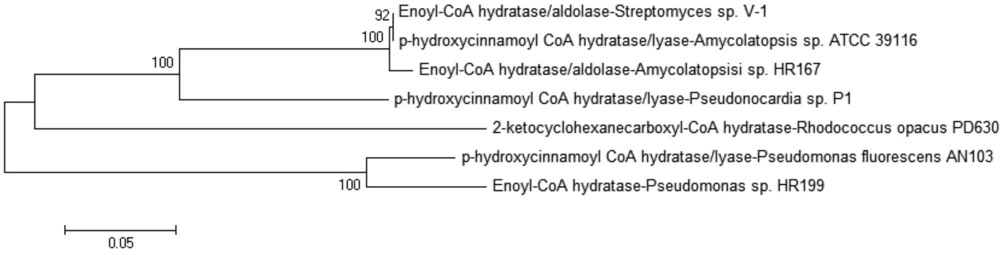

Figure S3. Homologies of enoyl-CoA hydratase/aldolase from *Streptomyces* sp. V-1 with the CoA hydratases/aldolases from different organisms. A. The amino acid sequence of enoyl-CoA hydratase/aldolase from *Streptomyces* sp. V-1 deduced from *ech* gene (*i*) was aligned to: the amino acid sequence of (*ii*) the *p*-hydroxycinnamoyl CoA hydratase/lyase from *Amycolatopsis* sp. ATCC 39116 [8], (*iii*) the enoyl-CoA hydratase/aldolase from *Amycolatopsis* sp. HR167 [10], (*iv*) the

p-hydroxycinnamoyl CoA hydratase/lyase from *Pseudonocardia* sp. P1 (unpublished data), (*v*) the 2-ketocyclohexanecarboxyl-CoA hydratase from *Rhodococcus opacus* PD630 [29], (*vi*) the *p*-hydroxycinnamoyl CoA hydratase/lyase from *Pseudomonas fluorescens* AN103 [13], and (*vii*) the enoyl-CoA hydratase from *Pseudomonas* sp. HR199 [7]. Amino acids are specified by standard one-letter abbreviations. *Dashes* indicate gaps introduced into the sequences to improve the alignment. **B.** The relationship between the enoyl-CoA hydratase/aldolase from *Streptomyces* sp. V-1 and the proteins in panel **A** was displayed as a phylogenetic tree, which was constructed on the basis of the Neighbor-Joining (NJ) method using the ClustalX and MEGA 5.0 softwares. The bar indicates 10% difference in amino acid sequence. The number at the branch point represents the percentage of 1,000 bootstrap repetitions.
