# Supplementary material for: Characterization of Two Streptomyces Enzymes That Convert Ferulic Acid to Vanillin
Source: PLoS One. 2013 Jun 28;8(6):e67339. doi: 10.1371/journal.pone.0067339 (PMC3696112; doi:10.1371/journal.pone.0067339)
Supplement: Figure S4 — GC spectrum of the conversion from ferulic acid to vanillin by both Fcs and Ech. The three lines indicate samples obtained at 0 min (blue), 5 min (red), and 10 min (green) during the enzymatic reactions with two purified proteins Fcs and Ech in the reaction mixture. (PDF) [file pone.0067339.s004.pdf]

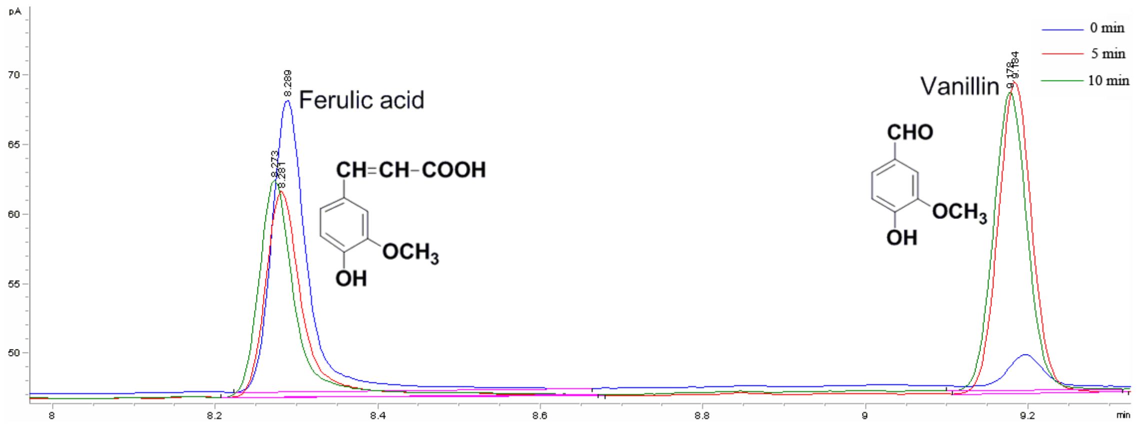

Figure S4. GC spectrum of the conversion from ferulic acid to vanillin by both Fcs and Ech. The three lines indicate samples obtained at 0 min (blue), 5 min (red), and 10 min (green) during the enzymatic reactions with two purified proteins Fcs and Ech in the reaction mixture.
