# Supplementary material for: Characterization of Two Streptomyces Enzymes That Convert Ferulic Acid to Vanillin
Source: PLoS One. 2013 Jun 28;8(6):e67339. doi: 10.1371/journal.pone.0067339 (PMC3696112; doi:10.1371/journal.pone.0067339)
Supplement: Figure S5 — Determination of the kinetic constants of Fcs. Michaelis-Menten and Lineweaver-Burk reciprocal plots of Fcs were determined by changing the concentration of the substrate (ferulic acid) from 0.175 mM to 0.7 mM. Values represent the means of three independent experiments; the error bars represent standard deviations. (PDF) [file pone.0067339.s005.pdf]

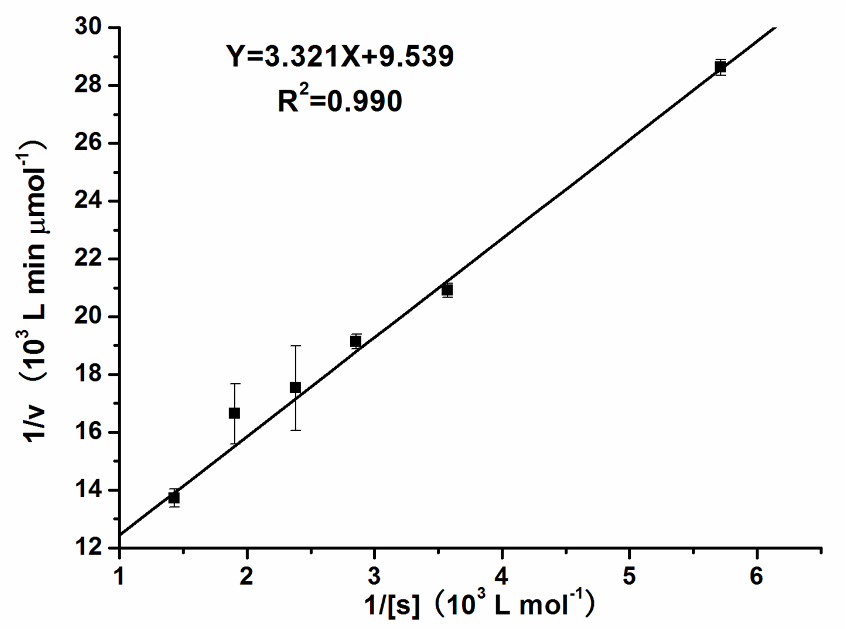

Figure S5. Determination of the kinetic constants of Fcs. Michaelis-Menten and Lineweaver-Burk reciprocal plots of Fcs were determined by changing the concentration of the substrate (ferulic acid) from 0.175 mM to 0.7 mM. Values represent the means of three independent experiments; the error bars represent standard deviations.
