# Supplementary material for: Characterization of Two Streptomyces Enzymes That Convert Ferulic Acid to Vanillin
Source: PLoS One. 2013 Jun 28;8(6):e67339. doi: 10.1371/journal.pone.0067339 (PMC3696112; doi:10.1371/journal.pone.0067339)
Supplement: Table S1 — The amino acid sequence similarities of Fcs and Ech in different organisms. (DOC) [file pone.0067339.s006.doc]

**Table S1.** The amino acid sequence similarities of Fcs and Ech in different organisms.

| Organism | Fcs (*Streptomyces* sp. V-1) Identity (%) | Ech (*Streptomyces* sp. V-1) Identity (%) |
| --- | --- | --- |
| *Amycolatopsis* sp. HR167 | 100.0 | 99.0 |
| *Amycolatopsis* sp. ATCC 39116 | 99.8 | 100.0 |
| *Streptomyces hygroscopicus* subsp. jinggangensis 5008 | 60.1 | / |
| *Streptomyces* sp. e14 | 59.8 | / |
| *Streptomyces* sp. SCC 2136 | 58.6 | / |
| *Pseudomonas* sp. HR199 | 17.2 | 51.6 |
| *Pseudonocardia* sp. P1 | / | 78.7 |
| *Rhodococcus opacus* PD630 | / | 62.6 |
| *Pseudomonas fluorescens* AN103 | / | 59.2 |

/: the alignment was not performed.
